# Wellbeing as a policy framework for health promotion and sustainable development

**DOI:** 10.1093/heapro/daab066

**Published:** 2021-12-12

**Authors:** J Hope Corbin, Faten Ben Abdelaziz, Kristine Sørensen, Mihály Kökény, Rüdiger Krech

**Affiliations:** 1 Department of Health and Community Studies, Western Washington University, Bellingham, WA, USA; 2 Enhanced Wellbeing, Division of Universal Health Coverage and Healthier Populations, Department of Health Promotion, World Health Organization, Geneva, Switzerland; 3 Global Health Literacy Academy, Risskov, Denmark; 4 Global Health Centre, The Graduate Institute for International and Development Studies, Geneva, Switzerland; 5 Division of Universal Health Coverage and Healthier Populations, Department of Health Promotion, World Health Organization, Geneva, Switzerland

**Keywords:** wellbeing, resilience, sustainable development goals, social policy, public policy

## Abstract

For years Gross Domestic Product (GDP) has served as a key indicator of human progress and “successful” societies. Unfortunately, GDP has failed to robustly capture the human experience or predict resilience through crises; and obscures the presence of inequity -- a key determinant of suffering. It is clear the global community needs a new organizing principle: one that envisions and measures progress by focusing on the conditions that support health, resilience, and overall wellbeing. This paper examines key health promotion concepts and approaches, juxtaposed with philosophical underpinnings of the concept of wellbeing, alternative measurement strategies, and examples of wellbeing policy initiatives. In doing so, the paper highlights the relevance of wellbeing policy frameworks to health promotion, the utility of health promotion strategies for implementing wellbeing policy frameworks, and controversies and pitfalls that require consideration. The paper concludes by outlining how health promotion is uniquely poised to contribute to wellbeing policy frameworks that promote the sources of human and planetary thriving through sustainable development, and that promoting a wellbeing agenda can strengthen efforts to promote health by addressing social determinants and ensuring universal access to resources that support coping with emerging challenges and strengthen resilience.

The notion of health put forward in the WHO constitution describes a positive concept—‘a state of physical, mental and social wellbeing’—that is grounded in human rights, acknowledges the role of social conditions, inequity, and the two-way relationships between peace and security, and human and planetary harmony. It highlights the shared value of health and protection from common dangers and considers life-course development and the realities of changing environments. Notions of participation, shared responsibility, partnership, collective action and adequate health and social measures are situated as key objectives at the societal level. The full definition reads:

Health is state of complete **physical, mental and social well-being** and not merely the absence of disease or infirmity. The enjoyment of the highest attainable standard of health is one of the fundamental **rights** of every human being **without distinction of race, religion, political belief, economic or social condition**. The health of all peoples is fundamental to the attainment of **peace and security** and is dependent upon the **fullest co-operation of individuals and States**. The achievement of any State in the promotion and protection of health is of **value to all**. **Unequal development in different countries in the promotion of health and control of disease, especially communicable disease, is a common danger**. Healthy development of **the child** is of basic importance; the ability **to live harmoniously in a changing total environment** is essential to such development. The extension to all peoples of the benefits of medical, psychological and related knowledge is essential to the fullest attainment of health. **Informed opinion and active co-operation on the part of the public** are of the utmost importance in the improvement of the health of the people. **Governments have a responsibility for the health of their peoples which can be fulfilled only by the provision of adequate health and social measures.** (WHO, 1946; emphasis added)

This definition is meaningful to examine at this moment in time as it highlights the common value of health and common danger of disease faced across society—as we are witnessing with COVID-19. It also highlights the importance of ‘informed opinion’ which speaks to health literacy in our current era of informational and technological challenges (Van den Broucke, 2020), even if these were unimaginable at the time the definition was written. There is also a clear imperative that creating policies to promote health and wellbeing is a core responsibility of governments. Therefore, part of the work of promoting health, is providing policy guidance for health and wellbeing governance. 

For years, the field of health promotion has sought innovative approaches to meet these mandates. The Ottawa Charter operationalizes this work and centers notions of wellbeing:

To reach a state of complete physical mental and social wellbeing, an individual or group must be able to **identify and to realize aspirations, to satisfy needs, and to change or cope with the environment**. Health is, therefore, seen as **a resource for everyday life**, not the objective of living. Health is a positive concept emphasizing **social and personal resources, as well as physical capacities**. Therefore, health promotion is not just the responsibility of the health sector, but goes **beyond healthy lifestyles to wellbeing.** (WHO, 1986; emphasis added)

The term ‘wellbeing’ is referenced in the above quotes but not explicitly defined. In truth, core philosophical conceptions of psychological wellbeing are present. The notion of identifying and realizing aspirations relates directly to the Aristotle’s notion of *eudaimonia* which conceives of wellbeing as it relates to people’s ability to reason and live a meaningful life according to their own aims (Waterman, 1993; Camfield and Skevington, 2008; Ryff and Singer, 2008). Huta and Waterman describe eudaimonia, commonly translated as ‘flourishing’, as a principal focused on ‘activity reflecting virtue, excellence, the best within us, and the full development of our potentials [(Huta and Waterman, 2014), p. 1427]’. Panhcheva *et al.* describes how this conception draws upon key elements of developmental, clinical, existential and humanistic psychological theories and highlight six distinct dimensions: autonomy, environmental mastery, personal growth, relationships with others, purpose in life and self-acceptance (Pancheva *et al.*, 2020). The notion of environmental mastery connects directly with the Ottawa Charter phrasing regarding people’s ability to ‘change or cope with the environment’.

The Ottawa Charter also identifies nine prerequisites for health, which include: peace, shelter, education, food, income, a stable eco-system, sustainable resources, social justice and equity. These social determinants of health have a profound impact on wellbeing. As Fisher (Fisher, 2019) argues in his theory of public wellbeing the ‘contingent nature of wellbeing within contemporary social environments’ requires tending to the compounding and intersectional influence of social, economic, environmental and cultural stressors on people’s ability to experience and exercise wellbeing.

The ability of people to ‘change or cope with the environment’, social determinants, as well as the understanding of health as a ‘resource for everyday life’ align directly to a highly cited modern definition of wellbeing crafted by Dodge *et al.* (Dodge *et al.*, 2012), which describes wellbeing as a balance between the resources a person has access to and the challenges they face in their daily lives (Dodge *et al.*, 2012). The resilience created by an individual or group’s ability to leverage existing resources (both generalized and specific) to overcome stressors is the key tenet of one of health promotion’s central theories: salutogenesis (Mittelmark *et al.*, 2016).

## MEASURES AND POLICIES FOR WELLBEING

The Ottawa Charter begins by centering individuals and groups but quickly lifts the discussion to social and policy imperatives: ‘health promotion is not just the responsibility of the health sector but goes beyond healthy lifestyles to wellbeing’.

Unfortunately, the central tool used to track development for policy purposes—Gross Domestic Product (GDP)—is of limited utility as an indicator of human progress, especially from a health promotion perspective. First, GDP is a strictly economic measure, the health and wellbeing of people, society and the environment do not enter the equation. Inequity escapes scrutiny when all we measure is financial growth as an average—the consolidation of wealth among the rich or increases in numbers of people experiencing poverty are not detected (Rijpma *et al.*, 2017). Non-marketed goods, such as food produced in family gardens, is not captured. We also fail to notice the toll unbridled production takes on planetary resources and the degradation of environmental conditions. GDP is also not designed to evaluate *how* a profit is made or whether it was earned through the selling of addictive products, or services to help cope with depression, or to promote or mitigate sources of human suffering (Boarini and D’Ercole, 2013).

Nations, organizations, and scholars have been pointing out the inadequacy of GDP as the only measuring stick for human development for many years (Stiglitz *et al.*, 2009; Boarini and D’Ercole, 2013; Kubiszewski *et al.*, 2013; Jones and Klenow, 2016; Rijpma *et al.*, 2017). However, the urgency of this work is brought into stark relief during the COVID-19 pandemic.

GDP has proved useless for predicting resilience in a crisis. Some of the richest nations in the world have seen the worst outcomes during the pandemic (Chaudhry *et al.*, 2020). The inequity GDP renders invisible has been put on display as the most devastating impacts of the disease have been borne by the most vulnerable groups within those wealthy countries (Shadmi *et al.*, 2020). Black, Indigenous, and people of color, immigrant communities, and older adults are all groups experiencing greater rates of infection and death (Abrams and Szefler, 2020). The social determinants that contribute to NCDs are wreaking compounding damage during this crisis since preexisting conditions cause complications during COVID-19 infection. There are greater barriers in accessing medical care due to COVID-related lockdowns and/or fear of infection (Leitch *et al.*, 2020). People are suffering from social isolation and related mental health challenges. People living in inadequate and or crowded housing are seeing increased levels of exposures to infection, but even with greater risk of infection, maybe spending more time in unhealthy indoor environments taking an additional toll on their health (D’alessandro *et al.*, 2020).

It is clear the global community needs a new organizing principle–one that envisions, plans and measures human progress with a focus on the origins of health and resilience. There is an opportunity and urgency for health promoters to examine the sources of human and planetary thriving to expand notions of human development in directions that create hardy and sustainable societies, which are better poised to cope with stressors and ensure wellbeing.

### Promising measures

Looking at wellbeing at the societal level, Nussbaum identifies 10 key capabilities to support human flourishing: life, bodily health, bodily integrity, senses, imagination and thought, emotions, practical reason, affiliation, other species, play, and control over one’s political and material environment (Nussbaum, 2013). Her work, along with Sen and others, has spurred efforts to measure and proactively plan policy that broaden ways of assessing human development. The Human Development Index, developed by Mahbub ul Haq, and based on the capabilities approach, is a composite measure drawing together health, education and economic indicators (Kubiszewski *et al.*, 2013).

The OECD has also been working to develop measurements and policy guidance. The OECD policy framework for wellbeing (OECD, 2020) identifies key dimensions of current wellbeing including income and wealth, employment (including quality), housing, health, knowledge and skills, environmental quality, subjective wellbeing, safety, work-life balance, social connections and civil engagement. Measurement of these dimensions take into account considerations such as averages, inequity between groups, inequity at the extremes and deprivations. Further, the framework considers future resources for wellbeing including stocks, flows, risk factors and resilience in the realms of natural, human, economic and social capital. The OECD How’s Life report disseminates findings on wellbeing in OECD countries for more than 80 indicators of wellbeing, inequity and future resources (OECD, 2020).

WHO has been working since the 1990s to measure subjective assessments of quality of life at the individual level, with the WHOQOL tool (WHO, 1998) and has been progressing research to better assess the impact of the social determinants of health and wellbeing.

### Wellbeing policy initiatives

Around the world there is a gathering movement toward proactive policy initiatives intended to promote wellbeing at the societal level. For instance, Scotland’s National Health and Wellbeing Framework is a detailed plan for requiring intersectoral action between health and social sectors including specific requirements for integrated planning, implementation, and reporting (Scottish Government, 2014). The OECD has created the ‘Economy of Wellbeing’ which intends to provide opportunities for people to pursue their aspirations, expand opportunities to lift the most neglected, reduce inequity and promote ecological and social sustainability (*The Economy of Well-Being—OECD*, n.d.). New Zealand also began taking wellbeing into account in its financial planning by introducing its Wellbeing Budget, which prioritizes spending to support mental wellbeing, reduce child poverty, increase Maori and Pacific incomes, transition to a low-carbon emissions economy, and boost economic productivity (New Zealand Government, 2019). The United Arab Emirates National Programme for Happiness and Wellbeing has three key areas of action focused on including happiness policies, programs and services at every level of government and in workplaces, promoting wellbeing and happiness in the community and developing benchmarks and tools to measure wellbeing and happiness outcomes (UAE Government, 2016). The nation of Bhutan has been working for decades to create Gross National Happiness among its citizens by delivering on its four pillars: sustainable and equitable social and economic development, environmental conservation, cultural preservation and promotion, and good governance (Centre for Bhutan Studies and GNH, 2004). These Bhutanese efforts helped infuse notions of happiness and wellbeing into the development agenda through UN Resolution 56/309 and by laying some groundwork for the creation of UN Sustainable Development Goals (SDGs).

The SDGs advocate for action to create the conditions conducive to wellbeing. By eliminating poverty and hunger, improving health, ensuring quality education, equity, clean water, clean energy, decent work, responsible consumption and production, climate action, the protection of life below water and on land, and peace and justice—all through intersectoral partnership– governments could create societies that thrive.

These initiatives reflect core health promotion commitments. They engage multiple sectors. They follow through on commitments to human rights, and equity. They seek to create the social conditions required to enable all people to realize aspirations, meet their needs, and to build resilient people, communities and societies. The creative approaches represented by these initiatives work to guarantee intersectoral collaboration, participation, and investment in health and wellbeing outcomes.

## WELLBEING POLICY AS SALUTOGENIC

For all of the initiatives described above, the policy framework of wellbeing provides a rallying cry for multisectoral collaboration. Wellbeing motivates partners, harnesses funding and provides a clear sense of purpose. Perhaps a wellbeing policy framework can provide a collective sense of coherence in the true Antonovsky meaning (Eriksson, 2016). Antonovsky defined sense of coherence as:

…a global orientation that expresses the extent to which one has a pervasive, enduring though dynamic feeling of confidence that (1) the stimuli deriving from one’s internal and external environments in the course of living are structured, predictable, and explicable; (2) the resources are available to one to meet the demands posed by these stimuli; and (3) these demands are challenges, worthy of investment and engagement. [(Antonovsky, 1987), p. 19]

As discussed to this point, wellbeing policy, while working to reduce inequity overall, also seeks to ensure people have access to the social, economic and environmental resources needed to cope with stressors and thrive. These resources fall under the categories of generalized resistance resources (e.g. social capital, schools, employment etc.) and specific resistance resources (e.g. relevant social services and health care) (Eriksson, 2017) also in line with salutogenic theory. Universal access to these resources and a clearly communicated collective intention to make them available for everyone can contribute to a sense of coherence at individual and societal levels by providing that ‘global orientation’ that people, communities and nations can overcome stressors and threats. Societies that meet the basic needs of their constituents can create environments that are more structured, predictable and explicable. While the global community will still face unknown threats, people and nations that live in environments with baseline wellbeing can comprehend (health and other kinds of literacy), manage, and find meaning in events as they proactively respond and prevent them. These aspects of sense of coherence are clearly encompassed in eudemonic notions of wellbeing. Because people in a wellbeing-oriented society enjoy a good quality of life, community members and leaders alike have confidence that they have the resources necessary to meet challenges and have the sense that they should come together to face them to help return society to equilibrium.

## CONTROVERSARIES, CAUTIONS, QUESTIONS FOR CONSIDERATION

Policy frameworks that center wellbeing are not without controversies and pitfalls. First, there is controversy over which measures of wellbeing are most appropriate to serve as benchmarks for policy. There are issues of reliability and validity in both of subjective and objective measures of wellbeing. For instance, measures of subjective wellbeing are vulnerable to the mood of the respondent and their temperament (Diener, 2006).

Second, while wellbeing calls for a shift from centering economic valuations of progress (e.g. GDP), economic logic runs deep in current policy making. Price (Price, 2017) in her critique of UK policy efforts, makes the case that policy under the banner of ‘wellbeing’ might distort issues that require broad societal-level solutions into narrow frames citing the example of attempts to address mental health needs to improve ‘wellbeing’ rather than addressing poverty (because mental health services are less costly to provide than investments in reducing poverty). It is vital that attempts to improve wellbeing do not deepen inequity by improving conditions for privileged groups in society while leaving others behind.

Another pitfall of the GDP-oriented mindset is one could argue that the market offers individuals a venue for achieving wellbeing through exercise of choice and consumption of goods. This notion is popular although clearly problematic given rampant economic inequity, the failure of material goods to actually satisfy eudemonic notions of wellbeing, the hedonic treadmill (wherein people experience less wellbeing as an adaptation to more consumption (Diener *et al.*, 2006)) and the impacts to planetary wellbeing that unbridled consumption entails. A relate issue concerns the conflation of subjective wellbeing—which is difficult to measure and may be seen to have pharmacological solutions—with objective or social wellbeing (Atkinson, 2011). If it is not clear that we are building on a social model of health, there is a danger of drift toward individual responsibility for wellbeing as opposed to the kind of policy framework advocated for here. Going further, simultaneous work to promote wellbeing at multiple levels—international frameworks, national policy, social responsibility and at the institutional level—will likely be most effective (Frey and Stutzer, 2007; Bache and Reardon, 2016).

It is also important to reflect on the balance between meeting needs on one hand, with ensuring participation, engagement and the opportunity to influence the course of events as key aspects of wellbeing on the other. How can policy frames aimed at promoting wellbeing ensure that all people feel included in society and possess a sense of belonging toward a collective present and future within which they can engage?

A final question to reflect upon: would a focus on wellbeing distract from other core principles such as justice, democracy, peace, tolerance or is it possible to center these as we move forward from a health promotion perspective (Bache and Reardon, 2016)?

## THE WELLBEING AGENDA: HEALTHY PUBLIC POLICY FOR A POST-COVID WORLD

The Ottawa Charter defines the action areas for health promotion one of which is promoting healthy public policy. A wellbeing policy framework moves beyond advocating for health in all policies and updates the strategy to assume the protection and promotion of health under the broader umbrella of wellbeing. Health is still central to this conception, but it is better served by galvanizing policy that influences a greater number of its determinants. This wellbeing policy framework aligns conceptually with early WHO definitions of both health and health promotion. Health promotion practice can strengthen, deepen and increase the positive impact of the wellbeing agenda by harnessing our extensive knowledge base, practical tools, and the relevant experiences we have honed over nearly half a century. We can also provide guidance in addressing key controversies and pitfalls in wellbeing policy initiatives by relying on the foundational values of health promotion. To move the agenda forward, it will be important to begin multi-stakeholder dialogues to build a broader consensus base and to vet the pros and cons.
